# Children's Oral Health Status Among Urban and Rural Areas of Qassim Region, Saudi Arabia: A Cross-Sectional Study

**DOI:** 10.7759/cureus.47947

**Published:** 2023-10-30

**Authors:** Abdullah S Alhudaithi, Zeyad Alsughier, Hamad Alzaidan, Thiyezen A Aldhelai

**Affiliations:** 1 College of Dentistry, Qassim University, Buraydah, SAU; 2 Orthodontic and Pediatric Dentistry Department, College of Dentistry, Qassim University, Buraydah, SAU; 3 Orthodontic and Pediatric Dentistry Department, Faculty of Dentistry, Ibb University, Ibb, YEM

**Keywords:** urban, rural, qassim region, oral hygiene behavior, oral health status, children, awareness

## Abstract

Background: Dental caries is a disease that is quite common in children and has a negative impact on their oral health, mental health, and quality of life. This study aimed to collect and correlate information about oral health awareness, oral health status, and oral hygiene practices in the urban and rural areas of Saudi Arabia.

Methods: The cross-sectional study was carried out among three dental clusters of Qassim from November 2022 to April 2023 in 12 schools on seven- to 12-year-old children. Data collection was done using the WHO Oral Health Questionnaire for Children.

Results: The current investigation included 700 children, 360 males and 340 females. Both urban and rural parents were more educated. Most children in rural areas reported excellent gum health than in urban areas (48.2% and 41.3%, respectively). Pain was the most common cause of visiting the dentist in rural more than in urban areas (55.7% and 54.5%, respectively). A significantly higher frequency of sugar consumption was reported for rural children.

Conclusion: Most rural and urban children take care of their teeth. The vast majority of them use a toothbrush and toothpaste to clean their teeth. However, the dental visit was not regular and only related to the toothache. They need more oral health education and promotion programs to improve the knowledge of oral health behavior in the Qassim region and the rest of Saudi Arabia.

## Introduction

Diseases associated with oral health, such as dental caries, gingival bleeding, calculus, fluorosis, and oral mucositis, have been affecting humans for a very long time [1,2]. These diseases impose a significant financial burden on the individual patient and the existing healthcare system [3,4]. Dental caries in the primary dentition is a possible indicator of dental caries in the mixed dentition and permanent dentition later in life [5]. Evidence suggests that the prevalence of dental caries in Saudi Arabia was higher when compared with the international rates [6,7]. Oral lesions and manifestations, such as signs and symptoms of oral or systemic diseases, can negatively impact children and adolescents and reduce their quality of life [8,9]. The effects of poor oral health are multifactorial, affecting primary dentition and permanent tooth buds, restricting the growth and development of jaws, and influencing children psychologically and their quality of life [10,11].

Thus, it is widely understood that primary dentition lays the foundation for healthy permanent teeth. Hence, oral diseases in schoolchildren are recognized as a significant global concern [12]. To prevent and control dental diseases among school-aged children, the World Health Organization (WHO) recommends that schools implement oral health promotion programs [13].

Responsibility for one’s oral health practices should begin early, and children of school age need to do so. There is evidence that a more comprehensive knowledge of oral health, besides the application of the fundamentals of dental hygiene, can result in improved oral hygiene and a more positive perspective on dental health [1,14]. It is the shared responsibility of parents, educators, and dentists to instill in children a lifelong commitment to good oral hygiene practices. It is widely acknowledged that prevention is the cornerstone of contemporary dental practice [15]. The oral health literacy, awareness, and practices of school-aged children in Saudi Arabia have been evaluated as part of several research projects. Recent studies carried out in the Al Qassim region of Saudi Arabia have shown that the prevalence of oral disease has significantly increased both in urban and rural areas. This study also observed that as the number of children decreased in the family, the dietary practices and hygiene practices improved [14]. Kannan et al. [15] emphasized that oral health care education should be included in the school curriculum and that a parental awareness program is needed to emphasize their role in the dental health of their children [5,9,14,16]. However, there is a lack of data that correlates the rural population of Saudi Arabian schoolchildren with the urban population of Saudi Arabian schoolchildren. Therefore, the purpose of this study was to assess oral health awareness, oral health status, oral hygiene practices followed, and the correlation between these factors and the age of the children and the level of education of the parents.

## Materials and methods

Study design, setting, and participants

This cross-sectional study was carried out between November 2022 and April 2023 among three dental clusters of the Qassim region: Buraydah City, Al Bukayriyah, and Unaizah regions of Qassim province. A list of schools was derived from the Ministry of Education website. Twelve schools were randomly selected for sample collection. The study was carried out at twelve different schools, six in urban and six in rural sites. A total of 1000 schoolchildren were provided study proforma from the three locations mentioned above. Out of them, 700 subjects participated in the main study with a response rate of 70%. The questionnaire was handed out to the included study subjects from seven to 12 years in the schools. Students absent on the examination day or out of the previous criteria were excluded from the study. Prior to the beginning of the research project, written informed consent was collected from the participants’ parents. Furthermore, the study got ethical approval from the Institutional Review Board of Qassim Research Center (DRC), College of Dentistry, Qassim University (EA/6116/2021).

Data sources and measurement

A study-specific questionnaire, the WHO Oral Health Questionnaire for Children, was used for data collection [17]. It is used to collect information about children's gender, age, parental education level, oral health status (excellent, good, average, and poor), and oral health behaviors like tooth brushing with toothpaste (at least two, once daily or less), frequency of carbohydrate intake (at least once daily or less), and the number of dental visits (at least once daily or less) with the reason for their visits. Before the start of the survey, the questionnaire was translated into Arabic by the translation and back-translation method. An expert in the pediatric dentistry department at the College of Dentistry, Qassim University, did that. The Arabic version of the questionnaire was pilot-tested to assess the awareness, attitudes, and practices of 30 children. These data were included in the final analysis of our study. A questionnaire consisting of various demographic and clinical questions can be divided into three parts: knowledge, attitude, and practice related to oral hygiene practices, which was adapted from the WHO primary oral health survey for children. The investigator who worked on this study was well-trained and calibrated in the pediatric dentistry department.

This questionnaire was circulated among all participants through the Google Forms link and hard copy. Children from seven to 10 years old were helped by their parents to complete the questionnaire, while children from 11 to 12 completed it by themselves. The response was entered in an Excel sheet, and decoding was done using an independent investigator. 

Study size

The sample size was calculated using G Power software (http://www.gpower.hhu.de/en.html). Based on the power of previously published research and our study's expected power (0.80) and confidence interval of 95%, the lowest perception was taken for the calculation of sample size. Based on the effect size, 600 participants were required.

Statistical analysis 

All qualitative variables were presented using frequency and percent, while the quantitative variable (age) was presented using mean and standard deviation. Chi-square and independent t-tests were used to compare urban and rural groups. All tests were two-tailed, and the significance level was set at a p-value ≤0.05. Data were analyzed using IBM Statistical Package for Social Sciences (SPSS) version 23, Armonk, NY, USA.

## Results

The current investigation included a total of 700 children, including 360 males and 340 females. The proportion of male students attending urban schools was higher (51.6%) than those attending rural schools (51.3%). In contrast, the proportion of female students attending rural schools was higher (48.7%) than those attending urban schools (48.4%). However, there were no significant differences between the male and female groups. It was found that there was no significant difference in the distribution of children in the study regarding their age. The highest percentage of the parents (father and mother) did significantly finish their education at a college or university in urban and rural areas (67.6%, 65.4%, 67.5%, and 70.4%, respectively) (Table 1).

**Table 1:** Demographic profile of the participating children. *Statistically significant difference at p-value≤0.05.

Variables		Urban (n = 312)	Rural (n = 388)	Total (n = 700)	Test (p-value)
Sex: n (%)					
Male		161 (51.6%)	199 (51.3%)	360 (51.4%)	0.007 (0.940)
Female		151 (48.4%)	189 (48.7%)	340 (48.6%)	
Age in years: Mean ± SD		9.21 ± 1.94	9.43 ± 1.83	9.33 ± 1.89	1.537 (0.125)
Level of parent’s education: n (%)					
Father	No formal school	0 (0%)	6 (0.9%)	6 (0.9%)	14.665 (0.002*)
	Less than high school	6 (1.9%)	25 (6.5%)	31 (8.4%)	
	High school completed	69 (22.1%)	68 (17.5%)	137 (39.6%)	
	College/university completed	211 (67.6%)	262 (67.5%)	473 (67.6%)	
Mother	No formal school	1 (0.3%)	6 (1.5%)	7 (1%)	7.669 (0.053*)
	Less than high school	14 (4.5%)	31 (8.0%)	45 (6.4%)	
	High school completed	61 (19.6%)	60 (15.5%)	121 (17.3%)	
	College/university completed	204 (65.4%)	273 (70.4%)	477 (68.1%)	

The distribution of children's responses toward oral health status and dental visits is presented in Table 2, which shows how they feel about their health. During the evaluation of the category's responses, a significantly higher number of children from rural areas responded that they had excellent oral health for their teeth and gum (48.2% and 48.2%, respectively) than the urban children (41.3% and 40.4%, respectively). On the other hand, there was a significant decrease (p-value = 0.023) in responses to the category "good" for oral health teeth and gum from rural children than urban children (30.2-26.3% for teeth and gum health of rural and 39.1-37.8% for teeth and gum health of urban). A moderate response was collected from average categories among both groups. In contrast, only 22-17 children living in urban areas and 18-22 in rural areas reported poor oral health. When it came to whether the children had experienced toothache or discomfort in the previous 12 months, there were no statistically significant differences for the exact ordinary times. However, “occasionally” was chosen as the highest time among the other options (n = 230). This finding can be correlated with the following question, which measured the number of times an individual went to the dentist in the preceding year. The highest percentage for the number of dentist visits was at least once in the past for all urban and rural participants (35.6% and 36.6%, respectively). However, there was no statistically significant difference among the groups.

Most children reported “pain or trouble with teeth, gums, or mouth” as the most common reason for a dental visit (55.1%), with a slight increase in rural compared to urban areas. The “treatment and follow-up” reason was significantly higher in the rural section than in the urban area (21.1% and 15.7%, respectively). A considerable percentage of children in both groups went to the dentist for routine checkups and treatment of their teeth (15.1%). Moreover, the urban children reported a significantly higher response (n = 50) for remembering their most recent dentist visit than the rural children (n = 27).

**Table 2:** Distribution of child response according to oral health status and dental visits. *Statistically significant difference at p-value≤0.05.

Variables	Urban (n = 312)	Rural (n = 388)	Total (n = 700)	Test (p-value)
How would you describe the health of your teeth: n (%)				
Excellent	129 (41.3%)	187 (48.2%)	316 (45.1%)	9.511 (0.023*)
Good	122 (39.1%)	117 (30.2%)	239 (34.1%)	
Average	34 (10.9%)	57 (14.7%)	91 (13%)	
Poor	22 (7.1%)	18 (4.6%)	40 (5.7%)	
How would you describe the health of your gums: n (%)				
Excellent	126 (40.4%)	187 (48.2%)	313 (44.7%)	11.243 (0.010*)
Good	118 (37.8%)	102 (26.3%)	220 (31.4%)	
Average	33 (10.6%)	54 (13.9%)	87 (12.4%)	
Poor	17 (5.4%)	22 (5.7%)	39 (5.6%)	
How often during the past 12 months did you have toothache: n (%)				
Often	32 (10.3%)	46 (11.9%)	78 (11.1%)	6.975 (0.073)
Occasionally	94 (30.1%)	136 (35.1%)	230 (32.9%)	
Rarely	70 (22.4%)	89 (22.9%)	158 (22.7%)	
Never	105 (33.7)	94 (24.2%)	199 (28.4%)	
How often did you go to the dentist during the past 12 months: n (%)				
Less than once or never	96 (30.8%)	92 (23.7%)	188 (26.9%)	2.129 (0.345)
Once	111 (35.6%)	141 (36.3%)	252 (36%)	
Twice or more	98 (31.4%)	44 (11.3%)	209 (29.9%)	
What was the reason for your last visit to the dentist: n (%)				
Pain or trouble with teeth, gums, or mouth	170 (54.5%)	216 (55.7%)	386 (55.1%)	16.380 (0.001*)
Treatment/follow-up	49 (15.7%)	82 (21.1%)	131 (18.7%)	
Routine check-up	43 (13.8%)	63 (16.2%)	106 (15.1%)	
I don’t know/don’t remember	50 (16%)	27 (7.0%)	77 (11%)	

Table 3 shows the distribution of child response according to oral hygiene behaviors and sugar consumption. Although it was found that the highest percentage of cleaning teeth was for the children who cleaned their teeth once or more than twice per day for both groups (n = 216 and n = 226 children, respectively), with a slight increase in the rural children, it was not as significant a difference. When evaluating the use of oral hygiene aids, it was found that using a toothbrush and toothpaste was higher in the urban and rural sections, without any statistically significant difference. Similarly, 247 urban and 304 rural children were using fluoridated toothpaste, without any significant difference between the two groups.

**Table 3:** Distribution of child response according to oral hygiene behaviors and sugar consumption.

Variables	Urban (n = 312)	Rural (n = 388)	Total (n = 700)	Test (p-value)
How often do you clean your teeth: n (%)				
Never	19 (6.1%)	28 (7.2%)	47 (7.7%)	1.258 (0.739)
Less than once/day	100 (32.1%)	111 (28.6%)	211 (30.1%)	
Once/day	96 (30.8%)	120 (30.9%)	216 (30.9%)	
Twice or more/day	97 (31.1%)	129 (33.2%)	226 (32.3%)	
Do you use any of the following to clean your teeth or gums: n (%)				
Toothbrush	254 (81.4%)	303 (78.1%)	557 (79.6%)	3.361 (0.604)
Wooden toothpicks	12 (3.9%)	13 (3.4%)	25 (3.6%)	
Plastic toothpicks	4 (1.3%)	3 (0.8%)	7 (1.0%)	
Dental floss	15 (4.8%)	21 (5.4%)	36 (5.1%)	
Charcoal	9 (2.9%)	20 (5.2%)	29 (4.1%)	
Chewstick/miswak	18 (5.8%)	28 (7.2%)	46 (6.6%)	
Do you use toothpaste to clean your teeth: n (%)				
Yes	301 (96.5%)	379 (97.7%)	680 (97.1%)	0.906 (0.341)
No	11 (3.5%)	9 (2.3%)	9 (2.3%)	
Do you use toothpaste that contains fluoride: n (%)				
Yes	247 (79.2%)	304 (78.4%)	551 (78.7%)	1.401 (0.496)
No	14 (4.5%)	12 (3.1%)	26 (3.7%)	
I don’t know	51 (16.3%)	72 (18.6%)	123 (17.6%)	

Children’s perceptions in the past 12 months were recorded according to their oral hygiene status (Table 4). Although there were no significant differences among the groups, rural children seem to have more experience than urban children regarding these problems: appearance, smiling avoidance, toothache, and difficulty during biting and chewing. 

**Table 4:** Children’s perception based on their oral health status during the past 12 months.

Variables	Urban (n = 312)	Rural (n = 388)	Total (n = 700)	Test (p-value)
Because of the state of your teeth and mouth, have you experienced any of the following problems during the past year: n (%) (Yes answers)				
I am not satisfied with the appearance of my teeth	125 (40.1%)	147 (38.0%)	272 (38.9%)	0.154 (0.751)
I often avoid smiling and laughing because of my teeth	74 (23.7%)	82 (21.2%)	156 (22.3%)	0.404 (0.525)
Other children make fun of my teeth	30 (9.6%)	34 (8.8%)	64 (9.2%)	0.160 (0.689)
Toothache or discomfort caused by my teeth forced me to miss classes at school or miss school for whole days	41 (13.1%)	66 (17.1%)	107 (15.3%)	2.263 (0.132)
I have difficulty biting hard foods	53 (17.0%)	78 (20.2%)	131 (18.7%)	1.625 (0.202)
I have difficulty in chewing	43 (13.8%)	59 (15.2%)	102 (14.6%)	

Table 5 shows the distribution of child response according to the frequency of consumption of sugary food and drinks. It has been found that the usual consumption of fresh fruit and sweets or candy was at least once per day for both groups. It has been found that rural children have statistically higher biscuit/cakes/pies consumption per day (n = 118) than urban children (n = 57). Similarly, lemonade, Coca-Cola, and soft drinks consumption were statistically significantly higher in rural than urban children in the “less than once a day” category. On the other hand, the percentage of rural children who never take jam/honey and chewing gum was significantly lower than that of urban children (20.2%, 19.6%, 26.4%, and 27.4%, respectively). There was a significant increase in the rural children drinking milk and coffee with sugar than the urban children (n = 119 and n = 133, respectively), despite most children in both groups never drinking milk and coffee with sugar. There was a higher percentage of the “At least once/day” category in drinking tea with sugar for rural children than for urban children (62% and 51.9%, respectively).

**Table 5:** Distribution of child response according to frequency of consumption of sugary food and drinks. *Statistically significant difference at p-value≤0.05.

Variables	Urban (n = 312)	Rural (n = 388)	Total (n = 700)	Test (p-value)
Fresh fruits: n (%)				
Never	6 (1.9%)	14 (3.6%)	20 (2.9%)	4.202 (0.122)
Less than once/day	43 (13.8%)	69 (17.8%)	112 (16.0%)	
At least once/day	263 (84.3%)	304 (78.6%)	567 (81.1%)	
Biscuits/cakes/pies: n (%)				
Never	26 (8.3%)	32 (8.3%)	58 (8.3%)	14.136 (0.001*)
Less than once/day	57 (18.3%)	118 (30.5%)	175 (25%)	
At least once/day	229 (73.4%)	237 (61.2%)	466 (66.75)	
Lemonade/Coca-Cola/soft drinks: n (%)				
Never	60 (19.2%)	71 (18.3%)	131 (18.7%)	8.614 (0.013*)
Less than once/day	92 (29.5%)	154 (39.8%)	246 (35.2%)	
At least once/day	160 (51.3%)	162 (41.9%)	322 (46.1%)	
Jam/honey: n (%)				
Never	63 (20.2%)	102 (26.4%)	165 (23.6%)	6.385 (0.041*)
Less than once/day	90 (28.8%)	123 (31.8%)	213 (30.5%)	
At least once/day	159 (51%)	162 (41.9%)	321 (45.9%)	
Chewing gum: n (%)				
Never	61 (19.6%)	106 (27.4%)	167 (23.9%)	6.183 (0.045*)
Less than once/day	116 (37.2%)	137 (35.4%)	253 (36.2%)	
At least once/day	135 (43.3%)	144 (37.2%)	279 (39.9%)	
Sweets/candy: n (%)				
Never	20 (6.4%)	31 (8%)	51 (7.3%)	0.954 (0.621)
Less than once/day	70 (22.4%)	92 (23.8%)	162 (23.2%)	
At least once/day	222 (71.2%)	264 (68.2%)	468 (69.5%)	
Milk with sugar: n (%)				
Never	181 (58.2%)	175 (45.2%)	356 (51.0%)	11.915 (0.003*)
Less than once/day	61 (19.6%)	93 (24.0%)	154 (22.1%)	
At least once/day	69 (22.2%)	119 (30.7%)	188 (26.9%)	
Tea with sugar: n (%)				
Never	67 (21.5%)	84 (21.7%)	151 (21.6%)	11.877 (0.003*)
Less than once/day	83 (26.6%)	63 (16.3%)	146 (20.9%)	
At least once/day	162 (51.9%)	240 (62%)	402 (57.5%)	
Coffee with sugar: n (%)				
Never	181 (58.0%)	178 (46.0%)	359 (51.4%)	10.758 (0.005*)
Less than once/day	77 (24.7%)	133 (34.4%)	210 (30%)	
At least once/day	54 (17.3%)	76 (19.6%)	130 (18.6%)	

## Discussion

In order to aid in planning and evaluating national oral health programs, the current study was carried out to provide data on oral diseases and the oral health behaviors of children who attend school in rural and urban settings. The present study was conducted among seven- to 12-year-old children residing in urban and rural parts of the Qassim region. The availability of data correlating urban and rural populations was limited in Saudi Arabia, and it took much work to correlate with the local demographic profile.

Most parents in the present study completed degree courses and were found to be cautious about their children’s oral health. The level of education one has played a significant part in forming habits and the level of responsibility one takes for one’s health. The current study demonstrated that the percentage of fathers and mothers who completed the university study were 67.6% and 68.1%, respectively, almost near the results of Al Mejmaj et al. [5], Sabbagh et al. [9], and Alhareky et al. [18], with parental percentages of 79.3%, 66.0-67.7%, and 52.1-53.7%, respectively. 

In our research, most rural children seem more confident than urban children about what they think about the status of their gum and oral health, with the total percentage of children choosing excellent oral health among the two groups being 45.1%. These results come against Sabbagh et al. [9], in which only 25.3% of parents choose excellent oral health; this might be attributed to the fact that parents filled out the questionnaire, while in our research, it was done by children with their parent help for younger age children. On the other hand, urban children prefer to express their oral health condition with the term good more than rural children, according to previous research [9].

It was found by Sabbagh et al. [9] that about 32.1% of children suffer from toothache occasionally, which is almost similar to our results (32.9%). On the other hand, it was much higher in the research performed by Alhareky et al. [18] (46.8%). Also, in this study, most children from urban and rural environments (36%) reported that they visited the dentist at least once in the past 12 months. The same attitude was also found in the Qassim region with Prabhu et al.'s study [14] and in the Dammam region with Alhareky et al.'s research [18]. The pain or trouble of teeth and gum was the most significant common reason for rural and urban children to visit the dentist (55.7% and 54.5%, respectively). These results came in accordance with many studies conducted in different provinces and regions of Saudi Arabia [9,15,18-21]. Furthermore, our study was consistent with Rafi et al. [22], which reports that about 54.6% of rural children visit the dentist due to tooth pain.

The common frequency of cleaning teeth among urban and rural children was once or twice daily (63.2%). Similar results were found in different research in Saudi Arabia regions [14,18,20]. The use of toothbrush with fluoridated toothpaste in our study was higher in urban and rural children (79.6%, 97.1%, and 78.7%, respectively), with no significant difference between them, which was in alignment with the results observed by Prabhu et al. [14] with 69.0%. Similar results were also seen in Kannan et al. [15] study (72.7%). However, Rafi et al. reported that the percentage of rural children in southern Saudi Arabia who used a toothbrush with toothpaste for teeth cleaning was only 58.4% [22]. It might be because the participants were much older (10-18 years), and older children tend to be more careless about oral hygiene. It has been shown in Table 3 that the other cleaning methods were not used in a high number among the two groups of children, which is compatible with the other studies [14,15,21]. On the other hand, some researchers showed that the use of Miswak for teeth cleaning was slightly higher in male children of Jeddah (32.7%) [20]. Similarly, the percentage of children using Miswak in southern Saudi Arabia’s rural areas was 32.1% [22]. Only 6.6% of the urban and rural children of the Qassim region were reported using Miswak for oral cleaning. The reason behind that might be related to the great beliefs in Miswak in the community with religious bases. Since most participants in the previous two studies were adolescents, they tried to behave like adults, including their oral health habits.

In the present study, the children's perception of their oral health in the past 12 months was slightly increased in the rural children, with no significant difference between the two groups. About 38.9% of the rural and urban children were not satisfied with the appearance of their teeth. This percentage is similar to Alhareky et al. [18] (34.6%). Compared with the urban children, the rural children had pain in biting hard food (20.2%) and chewing (15.2%), in a slightly higher proportion, which confirms the previous study. 

Despite the facts, fresh and healthy fruits were consumed more often by participants in rural areas. The consumption of sweets was higher in rural children than in urban children (including biscuits, cake, pie, soft drinks, milk, coffee, and tea with sugar). However, rural children do not like to consume jam and chew gum compared to urban children. The high sugar consumption by rural children might explain why they have more problems biting and chewing than urban children. Furthermore, these results support previous results by Alhareky et al. [18]

Usually, the children depend on their parents to provide the necessary health care services [23]. In the current study, many parents from both groups showed a high educational level, which is one of the critical foundations of adequate oral health behaviors. It used to be that parents with higher education levels and socioeconomic status had more awareness of the family's oral health [24]. Thus, the parents can access all the dental services and follow the proper oral hygiene practices for them and their children. Even though there was a geographical and social difference between urban and rural areas, the children had access to all services while staying in a rural area. This could be because rural areas are often located in close proximity to urban areas and because oral health services are readily available and accessible. In the Qassim region, there was a previous recommendation regarding further studies to describe the children's oral health status as well as the dental knowledge of their parents [5]. In this study, the general level of the children and their parent's knowledge and behavior of oral health was reasonable, with a good tendency to improve oral health. However, there was a lack of proper knowledge and practice to improve their oral health. These data coincide with the previous study made in the Qassim region by Prabhu et al [14]. Although the perception of many oral health practices was similar between urban and rural children, there was some deficiency in children's oral health knowledge and oral health among the groups, especially in rural areas. This might be related to some environmental, social, and socioeconomic factors. The current results were found in many global comparable studies of oral health status between urban and rural children, as reported by studies on populations from Iran, Peru, Malawi, Thailand, India, Chile, Africa, and the Middle East Region [16,25-30].

One of the study's main limitations is that it was conducted in the Qassim region only; therefore, we cannot generalize our results to Saudi Arabia. Moreover, it relied on the observations of children, who may have been subject to information bias on the part of the informant and observer or may have made mistakes while filling out the survey; however, the questionnaire was pilot-tested for validity and calibrated before the application of the study to minimize this possibility of bias. Further studies might be needed, including a complete oral examination of different regions in Saudi Arabia. Furthermore, further research is needed to establish causative relationships between parental education and oral health practices. 

## Conclusions

The parental educational level was high in urban and rural areas of the Qassim region, which might reflect on their children's oral hygiene level. Most of the children included in this study had a reasonable amount of oral health knowledge and practices. There were some urban and rural disparities in using oral hygiene measures, especially with regular dental visits and the frequency of sugar consumption. The two groups' most common reason for dentist visits was dental pain. The current findings may guide the development of comprehensive dental education and dental promotion programs for parents and primary schools catering to rural and urban children.
